# Decoding Cancer through Silencing the Mitochondrial Gatekeeper VDAC1

**DOI:** 10.3390/biom14101304

**Published:** 2024-10-15

**Authors:** Tasleem Arif, Anna Shteinfer-Kuzmine, Varda Shoshan-Barmatz

**Affiliations:** 1 Sylvester Comprehensive Cancer Center, Miller School of Medicine, University of Miami, Miami, FL 33136, USA; 2 Department of Biochemistry and Molecular Biology, Miller School of Medicine, University of Miami, Miami, FL 33136, USA; 3 National Institute for Biotechnology in the Negev, Ben-Gurion University of the Negev, Beer-Sheva 84105, Israel; shteinfe@post.bgu.ac.il; 4 Department of Life Sciences, Ben-Gurion University of the Negev, Beer-Sheva 84105, Israel

**Keywords:** siRNA, metabolism, mitochondria, stem cells, VDAC1

## Abstract

Mitochondria serve as central hubs for regulating numerous cellular processes that include metabolism, apoptosis, cell cycle progression, proliferation, differentiation, epigenetics, immune signaling, and aging. The voltage-dependent anion channel 1 (VDAC1) functions as a crucial mitochondrial gatekeeper, controlling the flow of ions, such as Ca^2+^, nucleotides, and metabolites across the outer mitochondrial membrane, and is also integral to mitochondria-mediated apoptosis. VDAC1 functions in regulating ATP production, Ca^2+^ homeostasis, and apoptosis, which are essential for maintaining mitochondrial function and overall cellular health. Most cancer cells undergo metabolic reprogramming, often referred to as the “Warburg effect”, supplying tumors with energy and precursors for the biosynthesis of nucleic acids, phospholipids, fatty acids, cholesterol, and porphyrins. Given its multifunctional nature and overexpression in many cancers, VDAC1 presents an attractive target for therapeutic intervention. Our research has demonstrated that silencing VDAC1 expression using specific siRNA in various tumor types leads to a metabolic rewiring of the malignant cancer phenotype. This results in a reversal of oncogenic properties that include reduced tumor growth, invasiveness, stemness, epithelial–mesenchymal transition. Additionally, VDAC1 depletion alters the tumor microenvironment by reducing angiogenesis and modifying the expression of extracellular matrix- and structure-related genes, such as collagens and glycoproteins. Furthermore, VDAC1 depletion affects several epigenetic-related enzymes and substrates, including the acetylation-related enzymes SIRT1, SIRT6, and HDAC2, which in turn modify the acetylation and methylation profiles of histone 3 and histone 4. These epigenetic changes can explain the altered expression levels of approximately 4000 genes that are associated with reversing cancer cells oncogenic properties. Given VDAC1’s critical role in regulating metabolic and energy processes, targeting it offers a promising strategy for anti-cancer therapy. We also highlight the role of VDAC1 expression in various disease pathologies, including cardiovascular, neurodegenerative, and viral and bacterial infections, as explored through siRNA targeting VDAC1. Thus, this review underscores the potential of targeting VDAC1 as a strategy for addressing high-energy-demand cancers. By thoroughly understanding VDAC1’s diverse roles in metabolism, energy regulation, mitochondrial functions, and other cellular processes, silencing VDAC1 emerges as a novel and strategic approach to combat cancer.

## 1. Overview

Cancer cells typically acquire a set of common characteristics that include uncontrolled proliferation, metabolic reprogramming, enhanced survival strategies, and resistance to apoptosis. These characteristics arise from alterations in key oncogenes and non-oncogenes that are crucial for cancer cell survival. Consequently, effective cancer therapy aims to either reverse these properties or exploit them as specific vulnerabilities of tumors.

The voltage-dependent anion channel 1 (VDAC1), a pivotal mitochondrial gatekeeper protein, plays a crucial role in regulating mitochondrial function. It orchestrates the metabolic and energetic interactions between the mitochondria and the rest of the cell and is integral to mitochondria-mediated apoptosis [1,2,3,4,5,6]. Positioned at the intersection of cellular metabolism, VDAC1 regulates both mitochondrial and cellular energy functions. It also participates in various physiological and pathological processes, including inflammation and immune responses. This review examines how targeting VDAC1 could influence high-energy demanding cancer cells. The innovative approach of silencing VDAC1 in cancer therapy is informed by a comprehensive understanding of its complex roles in metabolism, energy regulation, mitochondrial function, and cellular mechanisms.

## 2. VDAC1 Structure and Multifunctional Role

In mammals, three isoforms of VDAC (VDAC1, VDAC2, and VDAC3) have been identified. These isoforms share some structural and functional similarities, but also exhibit distinct differences as unique regulatory roles and interactions that influence cellular metabolism and survival [7,8,9]. While all three are expressed across various tissues, in most tissues, VDAC1 is typically expressed at higher levels than VDAC2 and VDAC3, thus, making it the most abundant and extensively studied isoform [7,8,10].

**Figure 1:**
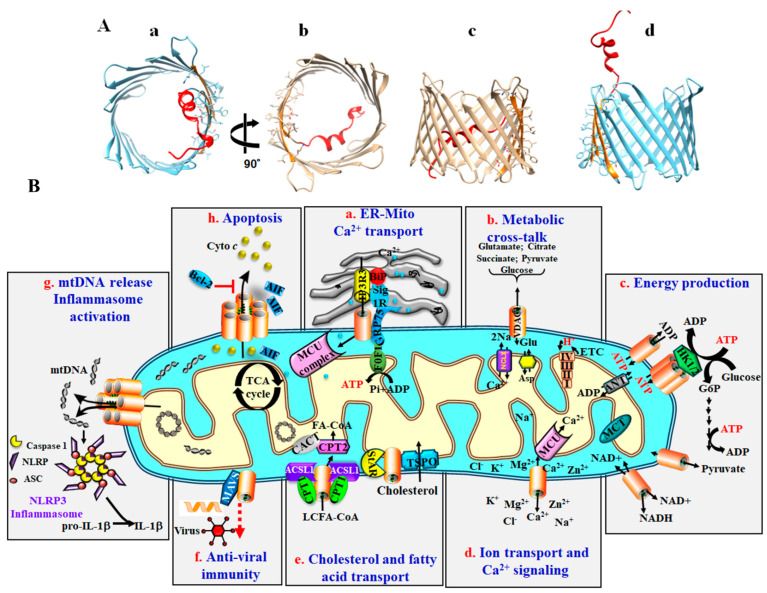
VDAC1 structure and its multi-functional protein involved in Ca^2+^ and metabolite transport, energy production, apoptosis, inflammation, and immune responses. (**A**) VDAC1 structure (PDB ID: 3EMN). VDAC1 structure; (**a**,**b**) top view, (**c**) side view and (**d**) side view with N-terminal α-helix exposed to the channel surface. (**B**) VDAC1, as a multifunctional channel, plays a crucial role in mediating mitochondrial interactions with the endoplasmic reticulum (ER) and cytosol. Its functions include: (**a**) ER–Mitochondria contacts: VDAC1 contributes to both the structural and functional contacts between the ER and mitochondria. It facilitates the transfer of Ca^2+^ released by inositol 1,4,5-trisphosphate receptors (IP3R) in the ER to the intermembrane space (IMS). This Ca^2+^ is then transported into the mitochondrial matrix by the Ca^2+^ uniporter (MCU) complex, where it regulates energy production through the activation of tricarboxylic acid cycle (TCA) enzymes such as pyruvate dehydrogenase (PDH), isocitrate dehydrogenase (ICDH), and α-ketoglutarate dehydrogenase (α-KGDH), as well as the electron transport chain (ETC) and ATP synthase (FoF1). (**b**) Metabolite transport: VDAC1 mediates the transport of metabolites up to 5 kDa between the mitochondria and the cytosol. (**c**) Energy production regulation: VDAC1 regulates energy production by facilitating the transport of ATP/ADP, NAD+/NADH, and acyl-CoA between the cytosol and IMS. It also binds to hexokinase (HK), channeling mitochondrially produced ATP directly to HK, which in turn regulates glycolysis. (**d**) Ions and Ca^2+^ homeostasis: VDAC1 controls the passage of ions and Ca^2+^ between the cytosol and IMS according to their concentration gradients, thus, maintaining Ca^2+^ homeostasis. (**e**) VDAC1 plays a crucial role in the transport of cholesterol, as it is a component of the multi-protein complex known as the transduceosome, which is responsible for cholesterol transport. VDAC1 is involved in the transport of long-chain fatty acids into the mitochondria through the carnitine shuttle, as part of the Acyl-CoA synthetase (ACSL1/5)/VDAC1/Carnitine Palmitoyl-Transferase 1A (CPT1a) complex located in the OMM. Long-chain fatty acids are activated to form acyl-CoA by ACSL1/5, which is then transferred across the OMM by VDAC1 into the IMS, where CPT1a converts the acyl-CoA into acyl-carnitine. This acyl-carnitine is subsequently transported across the inner mitochondrial membrane (IMM) via carnitine-acylcarnitine translocase (CACT) and is converted back into acyl-CoA by CPT2. Finally, the regenerated acyl-CoA can undergo β-oxidation in the mitochondrial matrix to produce energy. (**f**) Anti-viral immunity: VDAC1 interacts with the mitochondrial antiviral-signaling protein (MAVS) to enable antiviral signaling. (**g**) mtDNA releases inflammasome activation: VDAC1 oligomers facilitate the release of mitochondrial DNA (mtDNA), triggering the assembly of the inflammasome complex, including NLRP3, ASC, and caspase-1. This leads to the activation of NLRP3-dependent caspase-1 and conversion of pro-inflammatory factors such as pro-IL-1β to IL-1β. (**h**) Apoptosis: Upon apoptotic stimuli and stress conditions, VDAC1 is oligomerized, forming a hydrophilic protein-conducting channel that mediates the release of apoptogenic proteins, such as cytochrome c (Cyto c) and apoptosis-inducing factor (AIF), from the IMS to the cytosol, leading to apoptosis. Bcl-2 can interfere with the release of the pro-apoptotic proteins.

Structurally, VDAC1 is composed of 19 transmembrane β-strands connected by short flexible loops that form a β-barrel structure. Additionally, it features a 26-residue N-terminal region that extends into the pore [11,12,13,14] (Figure 1A). The N-terminal domain is able to exit the pore, as supported by studies showing that the N-terminal α-helix exhibits motion during voltage-gating [15,16,17,18]. Furthermore, anti-VDAC1 antibodies targeting the N-terminal region interact with membrane-bound VDAC1 [19,20]. When the N-terminal domain is exposed on the channel surface [18], it interacts with various proteins, including hexokinase (HK) [2,3,21,22,23,24,25,26,27], amyloid-β (Aβ) [28,29], and Bcl-2 and Bcl-xL [2,24,30,31,32,33,34]. Notably, cells expressing a truncated VDAC1, lacking the N-terminal segment, show resistance to apoptosis [30], highlighting the N-terminal domain’s crucial role in apoptosis induction. 

Purified and membrane-embedded VDAC1 can assemble into various oligomeric form, including dimers, trimers, tetramers, hexamers, and higher-order complexes [2,18,27,35,36,37,38,39,40,41,42]. The contact sites between VDAC1 molecules within dimers and higher-order oligomers have been identified [43].

The OMM acts as the critical interface between the cytosol and the mitochondrion, with VDAC1 regulating cell bioenergetics. Metabolites and ions cross the OMM to reach the mitochondrial intermembrane space (IMS) before being transported into the matrix by approximately 53 secondary transport proteins, known as mitochondrial carriers. These carriers, part of solute carrier family 25 (SLC25), are substrate specific and facilitate transport driven by electrochemical, chemical, and membrane potential gradients. The SLC25 family includes carriers for Pi (PiC) and ADP/ATP (ANT), as well as other substrates such as aspartate/glutamate pyruvate, acyl carnitine, oxoglutarate, and citrate [44]. In contrast, VDAC1 is the sole channel in the OMM responsible for mediating the flux of ions, nucleotides, and other metabolites up to ~5000 Da, including pyruvate, malate, succinate, and NADH/NAD, as well as heme and cholesterol [2,5] (Figure 1B). 

VDAC1 is strategically positioned as a gatekeeper for substrate and product transport between the mitochondria and cytosol. Situated at the OMM, VDAC1 serves as a central hub protein that interacts with a range of proteins that coordinate mitochondrial functions with various cellular activities [1,2,27,40,45,46,47]. By interacting with approximately 150 different proteins, it plays a crucial role in mediating and regulating the integration of mitochondrial functions and cellular processes [28,36,41,43,48,49,50].

VDAC1 is a component of the transduceosome, a multi-protein complex that also includes the high-affinity cholesterol-binding protein, translocator protein (TSPO) and the steroidogenic acute regulatory protein (STAR) [51]. Additionally, VDAC1 is part of another complex that facilitates the transport of long- or short-chain fatty acids across the OMM (Figure 1) [52]. This complex comprises long-chain acyl-CoA synthetases (ACSLs) located at the OMM and carnitine palmitoyltransferase 1a (CPT1a), which faces the IMS. In the proposed model, ACSL1/5 activates long-chain fatty acids to form acyl-CoA, which is then transferred across the OMM by VDAC1 into the IMS, where CPT1a converts acyl-CoA into acyl-carnitine. This acyl-carnitine is then transported across the inner mitochondrial membrane (IMM) by carnitine-acylcarnitine translocase (CACT) and is converted back into acyl-CoA by CPT2 [53]. The regenerated acyl-CoA can then enter β-oxidation in the mitochondrial matrix to generate energy.

In addition to its role in regulating cellular energy and metabolism, VDAC1 is essential for mitochondria-mediated apoptosis. It is involved in the release of apoptotic factors and interacts with anti-apoptotic proteins such as Bcl-2, Bcl-xL, and HK, which are often overexpressed in cancer cells [54,55].

Several mechanisms have been proposed to explain how the mitochondria mediate the release of pro-apoptotic proteins [2,25,27]. Studies from our group and others have shown that apoptosis induced by various agents—such as chemotherapy drugs, arbutin, prednisolone, cisplatin, viral proteins, elevated cytosolic Ca^2+^, or UV irradiation—results in increased VDAC1 expression levels [55]. This overexpression leads to VDAC1 oligomerization, forming large pores that facilitate the release of mitochondrial pro-apoptotic proteins [36,39,40,41,50,56,57]. Our studies further indicate that VDAC1 oligomerization is a dynamic process and a common mechanism across different apoptotic stimuli, acting through various initiating pathways [36,39,40,41,42,48,49,50,56,57,58]. Additionally, we have identified VDAC1-interacting molecules such as diphenylamine-2-carboxylate (DPC) [58], 4,4 diisothiocyanostilbene-2,2-disulfonic acid (DIDS), 4-acetamido-4-isothiocyanato-stilbene-2,2-disulfonic acid (SITS), 4,4′ diisothiocyanatodihy-drostilbene-2,2′-disulfonic acid (H2DIDS), 4,4′-dinitrostilbene-2,2′-disulfonic acid (DNDS) [58], and novel compounds developed in our lab, VBIT-4 and VBIT-12, which prevent VDAC1 oligomerization, and thereby mitigate apoptosis and mitochondrial dysfunction [56].

VDAC1 plays a key in the regulation of several inflammatory pathways. It modulates the release of pro-inflammatory cytokines including tumor necrosis factor-α (TNF-α), interlukin-1β (IL-1β), and interleukin-6 (IL-6) by affecting mitochondrial function and the cellular redox state [59]. Additionally, VDAC1 has been shown to interact with inflammasomes, particularly the NOD-, LRR-, and pyrin domain-containing protein 3 (NLRP3) inflammasome [60]. This interaction impacts the activation and secretion of IL-1β and IL-18, which are essential for driving inflammatory responses [60]. Recent studies from our group using animal models of inflammatory diseases such as arthritis and colitis have demonstrated that depleting or inhibiting VDAC1 reduced inflammation and alleviated other symptoms [61], suggesting its role in modulating inflammatory processes.

Additionally, VDAC1 affects various immune cells, including macrophages, T cells, and dendritic cells [59]. It plays a role in regulating immune cell activation, proliferation, and apoptosis. Alterations in VDAC1expression can affect T-cell metabolism and function, influencing both adaptive and innate immune responses [59]. Moreover, dysregulation of VDAC1 has been linked to autoimmune diseases [62]. For instance, alterations in VDAC1 expression or function have been associated with the pathogenesis of diseases such as lupus [62] and multiple sclerosis [63].

VDAC1 impact on various cellular processes makes it a critical target for therapeutic intervention in metabolic, inflammatory, and autoimmune diseases. Novel compounds that interact with VDAC1 and inhibit its oligomerization, such as VBIT-4 and VBIT-12, have demonstrated efficacy in mitigating the pathophysiology of several conditions, including type-2-diabetes [64], lupus [62], colitis [61], Alzheimer’s disease [65], acute liver injury [66,67], spinal cord injury [68], and COVID-19 [69]. VBIT-4 known to inhibit mtDNA release [62], protected against cGAS-STING activation during SARS-CoV-2 infection [70].

This review focuses on the role of VDAC1 at the crossroads of cellular metabolism and its involvement in various pathways that are crucial in cancer cell survival and progression.

## 3. VDAC1 Overexpression in Cancer

**Figure 2:**
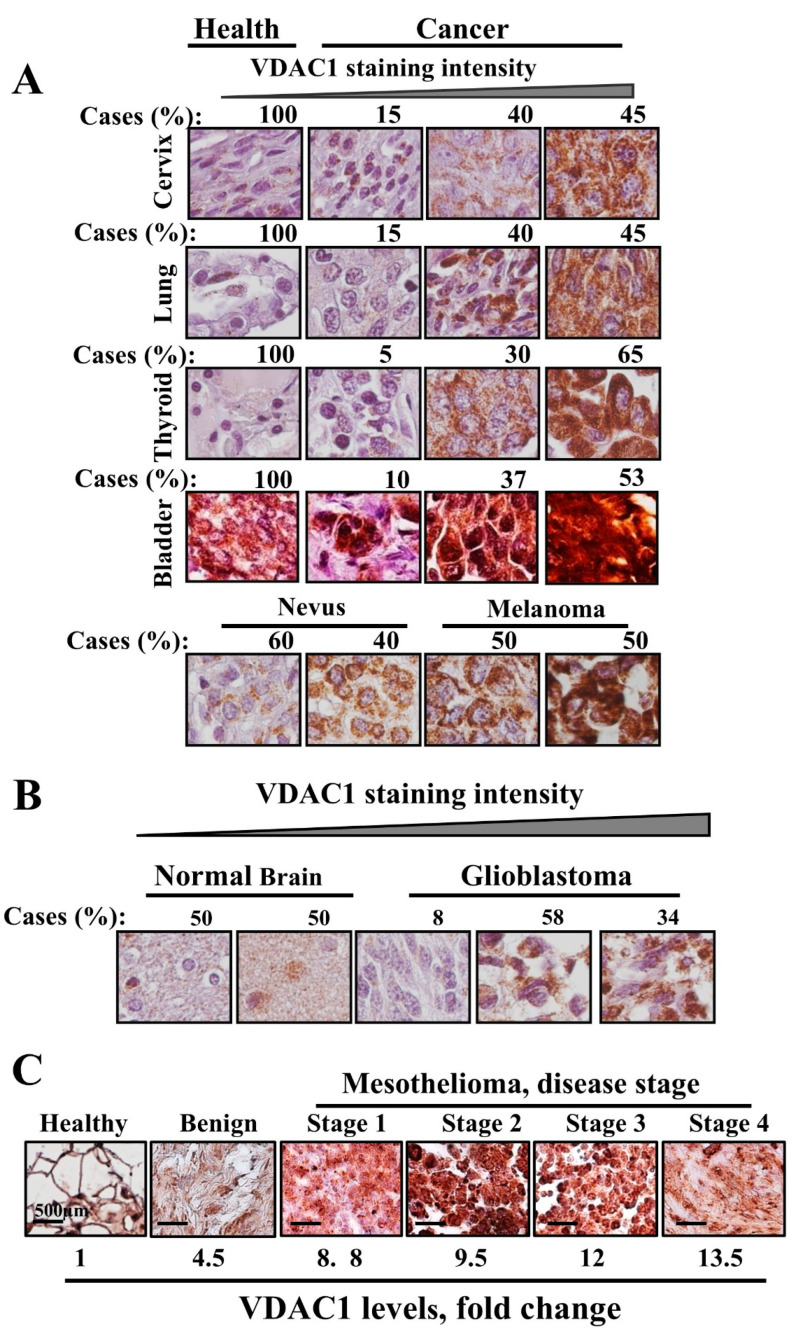
VDAC1 is overexpressed in different tumors. Cancer tissue arrays (Biomax), including sections from healthy and tumor tissues, subjected to immunohistochemistry (IHC) staining using anti-VDAC1 antibodies and representative tissue sections from cervical, lung, thyroid, bladder and melanoma (**A**), glioblastoma (**B**) and mesothelioma (**C**) cancers. Percentages of sections stained at the intensity indicated are depicted.

Cancer, characterized as a disorder of tissue proliferation, involves complex metabolic rewiring beyond mere growth and proliferation [71]. Cancer cells undergo profound metabolic changes, including significant alterations in glucose and glutamine metabolism, reflecting the plasticity of their metabolic machinery [72,73]. Our research has shown that VDAC1, a key player in cellular metabolism, is notably overexpressed across various cancer types compared to non-cancerous tissues. This was observed in tissue arrays for cancers including thyroid, lung, cervix, ovary, pancreas, melanoma, and glioblastoma, as well as in lung tissue samples from both healthy and tumor-affected areas of the same patient [1,74,75] (Figure 2). Similarly, overexpression of VDAC1 has been reported in other studies in breast, colon, liver, lung, pancreatic, and thyroid cancers [76] and in lung tumors [76]. Additionally, VDAC1 levels were elevated in peripheral blood mononuclear cells (PBMCs) from patients with chronic lymphocytic leukemia (CLL) compared to those from healthy individuals [77]. Interestingly, higher VDAC1 levels have also been linked to malignancies of the biliary tract [78] and in gastric cancer [79].

Cervical cancer patients who displayed high VDAC1 immunoreactivity have been shown to experience higher rates of recurrence and poorer overall survival compared to those with lower VDAC1 levels [80]. In addition, VDAC1 has emerged as a potential diagnostic marker for colorectal cancer [81] and gastric cancer [79].

In various cancer cell lines, VDAC1 expression levels were found to be elevated compared to those in a control fibroblast cell line [82,83]. Specifically, the levels of all three VDAC isoforms were significantly higher in ascites hepatoma cells compared to normal liver cells [84]. Moreover, both VDAC1 transcript and protein levels were elevated in the lung cancer cell line H358 relative to A549 cells [85]. Additionally, elevated VDAC1 expression has also been associated with cancer prognosis, tumor progression, and sensitivity to chemotherapy [82,83,86,87].

Given that VDAC1 plays a central role in cell metabolism, its enhanced expression provides a significant advantage to cancer cells. This VDAC1 overexpression in cancer is despite its involvement in apoptosis, where increased levels typically lead to apoptotic cell death [49]. However, in cancer, this pro-apoptotic effect is countered by the overexpression of anti-apoptotic proteins such as Bcl-2, Bcl-xL, and HK, which interact with VDAC1 [21,24,30,31,88].

## 4. VDAC1 Depletion Reduces Energy Conversion and Inhibits Cell Proliferation

RNA interference (RNAi) is a natural cellular process that targets specific mRNA molecules for degradation, thereby reducing the conversion of the corresponding protein [89,90]. Cellular metabolic and energy reprogramming are critical hallmarks of cancer, and VDAC1 plays a significant role in regulating these processes [2,5,27,38,91,92]. Downregulation of VDAC1 expression is anticipated to affect cancer cell growth.

Decreasing VDAC1 levels is expected to impair the exchange of metabolites between the mitochondria and the cytosol, leading to inhibited cell growth. In fact, using nanomolar (nM) concentrations of siRNA specific to human VDAC1 across 30 cell lines has shown that VDAC1 silencing reduces membrane potential (ΔΨ), lowers cellular ATP levels, and inhibits cancer cell proliferation without affecting non-cancerous cells [19,75], This suggests that the absence of VDAC1 impacts mitochondrial energy conversion.

Interestingly, while VDAC1 levels were reduced after the initial si-hVDAC1 (which targets human VDAC1) transfection, significant decreases in the expression of metabolism-related proteins such as glucose transporters (Glut-1), hexokinase (HK), glyceraldehyde-3-phosphate dehydrogenase (GAPDH), and lactate dehydrogenase-A (LDH-A) were only observed following the third and fourth transfections when compared to controls [93]. Additionally, the expression levels of the Kreb’s cycle enzyme citrate synthase (CS), mitochondrial electron transport complex IVc, and ATP synthase subunit 5a were also significantly diminished in si-hVDAC1-treated cells after the third and fourth transfections, reflecting changes in oxidative phosphorylation (OXPHOS) [93]. Therefore, depleting VDAC1 could offer a promising treatment strategy for various cancers by shifting cancer cells from a proliferative state to a non-dividing, differentiated state.

After silencing VDAC1 expression in cancer cells for an extended period of 10–15 days, significant reductions in glioblastoma cancer stem cell (GSC) markers were observed, accompanied by morphological changes indicative of differentiation into more mature, normal-like cells [74,94]. Glioblastoma cells, such as U-87MG, are typically hypodiploid. VDAC1 depletion led to differentiation into astrocyte- and neuron-like cells [74,94]. Similarly, in triple-negative breast cancer cells (MDA-MB-231), the levels of human epidermal growth factor receptor 2 (Her2), prolactin (PRL), estrogen receptor (ER), and progesterone receptor (PR) were elevated following VDAC1 depletion [93]. In A549 cells, a non-small cell lung carcinoma model representing partially differentiated alveolar epithelial type II (AT2) cells [95], VDAC1 depletion resulted in reprogramming to a more differentiated state, with cells resembling type I pneumocytes (AT1s) or more mature AT2 cells [94].

Finally, the cell differentiation driven by metabolic reprogramming due to VDAC1 depletion is mediated through transcription factors (TFs). Extended VDAC1 depletion resulted in significant alteration in the expression of the major TFs such as tumor protein p53 (p53), hypoxia-inducing factor 1α (HIF-1α), and c-myelocytomatosis (c-Myc) [74,93,94]. All are known to be regulators of metabolism and cell growth, proliferation, and differentiation [96].

These findings highlight the intricate effects of VDAC1 depletion on a network of key regulators of cell metabolism, guiding cancer cells toward differentiation. This reciprocal relationship that was observed suggests a link between reduced energy, decreased cell growth, and lower stem cell levels, leading to differentiation. This phenomenon has also been observed in tumor mouse models and their microenvironment (Section 6).

## 5. Silencing VDAC1 in Cancer Mouse Models

**Figure 3:**
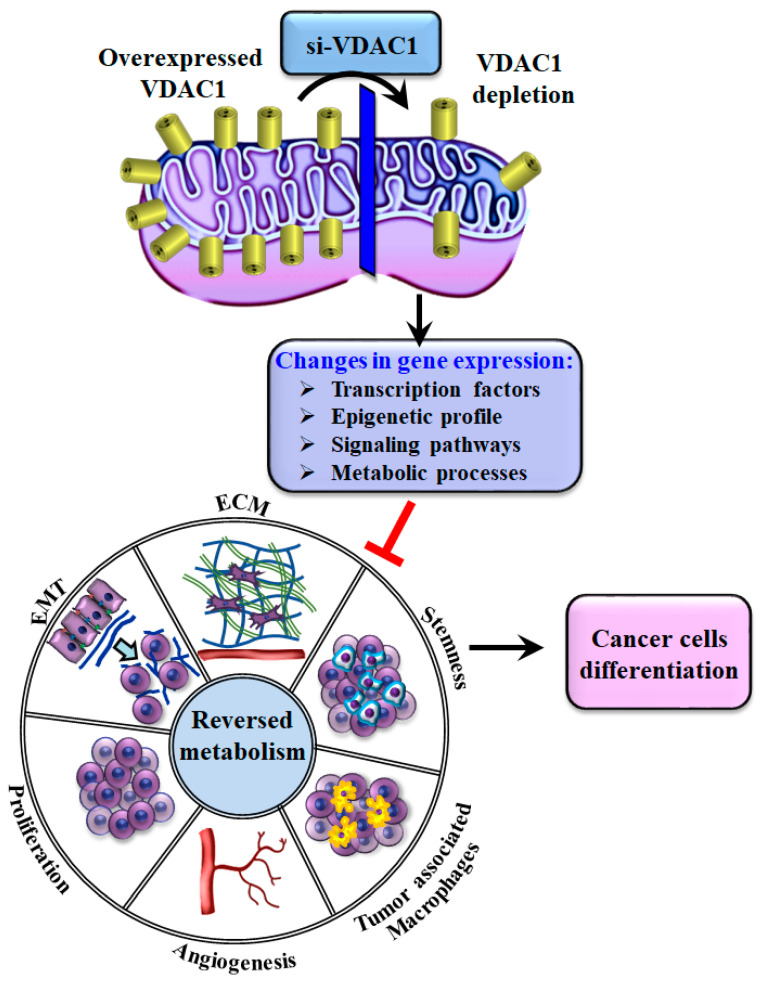
Overview of the effects of mitochondrial VDAC1 depletion on tumor properties: metabolic reprogramming and reversal of oncogenic properties. In cancer cells, overexpression of VDAC1 helps maintain energy and metabolic balance. Silencing VDAC1 disrupts this balance, leading to metabolic reprogramming that reduces energy production and metabolite generation critical for tumor growth and survival. This metabolic shift triggers changes in gene expression related to transcription factors (TFs), epigenetics, signaling pathways, the tumor microenvironment (TME), and stem cells. As a result, VDAC1 depletion impairs cell proliferation, remodels the TME, decreases angiogenesis, and reduces tumor-associated macrophages (TAMs). It also inhibits epithelial-to-mesenchymal transition (EMT), which is associated with increased cell migration and cancer progression and lowers the presence of cancer stem cells (CSCs) that are typically resistant to conventional therapies, while promoting the differentiation of cancer cells.

Several cancer mouse models have been utilized to highlight the critical role of VDAC1 in cancer survival and tumor growth. As detailed in Table 1, these models encompass lung, breast, liver, and bladder cancers, as well as mesothelioma and glioblastoma (GBM), with siRNA being administered either directly to the tumor or intravenously. The diverse effects of VDAC1 silencing in different cancer mouse models, including metabolic reprogramming and the reversal of tumor oncogenic properties, are illustrated in Figure 3.

### 5.1. Glioblastoma Multiforme (GBM)

In GBM, silencing VDAC1 led to significant changes in tumor characteristics [74]. In both subcutaneous and intracranial-orthotopic GBM models, treatment with si-hVDAC1 effectively inhibited tumor growth. The remaining tumor-like cells exhibited reversed oncogenic features, including altered metabolism, reduced angiogenesis, diminished EMT, and reduced invasiveness and stemness, as well as cell differentiation into neuron- and astrocyte-like phenotypes [74]. These VDAC1 depletion-mediated effects involved alterations in the expression of transcription factors (TFs) that regulate signaling pathways associated with cancer hallmarks, affecting the interaction between metabolism and oncogenic signaling networks, and leading to the differentiation of cancer stem cells (CSCs) into neuronal-like cells [74].

### 5.2. Lung Cancer

Lung cancer is the second most common cancer among both men and women and is responsible for 75–80% of cancer-related deaths, making it the leading cause of mortality worldwide [97]. This malignancy is influenced by a combination of genetic factors, including family history and polymorphisms, as well as environmental risks, with smoking being the primary risk factor [98,99]. Approximately 87% of lung-cancer cases are attributed to cigarette smoking due to its carcinogenetic properties [100].

Non-small cell lung cancer (NSCLC) is a leading cause of cancer-related deaths, accounting for 85% of cases. NSCLC comprises three main subtypes: adenocarcinoma (AC; 50%) [101], squamous cell carcinoma (SCC; 30%), and large cell carcinoma (LCC) [102]. AC typically occurs in the peripheral regions of the lung, while SCC is more commonly found centrally [103].

In our studies using various lung cancer cell lines, we found that siRNA targeting human VDAC1 with specific si-hVDAC1 led to decreased cellular ATP levels and inhibited cell proliferation [75]. In A549-derived tumors, silencing VDAC1 resulted in metabolic reprogramming, tumor regression, and significant alterations in the expression of thousands of genes, including TFs. This intervention also reduced CSCs, induced cell differentiation, and modulated the tumor microenvironment (TME), thereby disrupting tumor–host interactions [75,94,104].

Silencing VDAC1 expression was also effective in a chemically induced lung cancer model using the carcinogen urethane, which mimics the clinical scenario of lung cancer [105]. Treatment with si-VDAC1, which targets both mouse and human VDAC1, resulted in significant inhibition of VDAC1 expression levels. This led to reprogramming of cancer cell metabolism, a marked reduction in tumor growth for both NSCLC and small cell lung cancer (SCLC), and the elimination of CSCs. Similar outcomes were observed in SCLC xenografts [105]. These findings suggest that depleting VDAC1 could be a promising therapeutic strategy for treating both NSCLC and SCLC lung cancers.

### 5.3. Breast Cancer

Breast cancer, the most common malignancy among women in the United States, accounts for over 40,000 deaths annually and includes a diverse range of cancer cell phenotypes [106]. This cancer is categorized into two main subsets based on estrogen receptor (ER) expression: ER-positive and ER-negative breast cancers, each with distinct clinical characteristics [107,108,109]. Additionally, triple-negative breast cancer (TNBC) is a subtype that lacks expression of ER, PR, and the receptor tyrosine-protein kinase erbB-2 (ERBB2/Her2) [110,111].

The MDA-MB-231 cell line, derived from a metastatic pleural effusion and representing a poorly differentiated TNBC, does not express ER, PR, or ERBB2/Her2. However, following treatment with si-hVDAC1, either in MDA-MB-231 cells [93] or in tumors derived from these cells [94], there was an increase in the levels of ER, PRL, PR, and ERBB2/Her2. These changes in a breast cancer mouse model suggest differentiation into less malignant lineages. The increased expression of these receptors upon VDAC1 depletion highlights a potential avenue for Her2-based therapies for TNBC patients.

### 5.4. Bladder Cancer

Bladder cancer (BC) ranks as the tenth most frequently diagnosed malignancy globally, with over 400,000 new cases and nearly 200,000 deaths reported annually [112]. It is notably more common in men, and once metastasis occurs, the prognosis becomes significantly dire [113]. BC is the predominant malignancy of the urinary system, with urothelial carcinoma (UC), also known as transitional cell carcinoma, representing approximately 90% of cases. Other types of bladder cancer include squamous cell carcinoma, small-cell carcinoma, and adenocarcinoma [114]. UC is characterized by the invasion of neoplastic cells of urothelial origin into the basement membrane or lamina propria [115].

Using two mice models, one using subcutaneous UM-UC-3 cells and the other involving chemically induced BC with N-Butyl-N-(4-hydroxybutyl) nitrosamine (BBN), we demonstrated that suppressing VDAC1 expression had a significant impact on BC phenotypes [116]. Treatment with si-VDAC1 recognizing both murine and human VDAC1 (si-m/hVDAC1) led to metabolic reprogramming and subsequent inhibition of tumor growth [116]. Additionally, si-VDAC1 treatment resulted in modifications to the TME, including decreased angiogenesis, changes in the extracellular matrix (ECM), reduced tumor-associated macrophages, and inhibited stemness and EMT. These findings highlight the potential of si-VDAC1 as a promising therapeutic approach for BC [116].

### 5.5. Mesothelioma

Malignant mesothelioma is a rare, but highly aggressive cancer originating from the mesothelium, the tissue layer that lines and protects the serous cavities and internal organs, including the pleural cavity, peritoneum, pericardium, and tunica vaginalis testis [117,118]. Asbestos exposure is its primary cause, responsible for 80% of cases. Despite the rise in reported incidence over the past two decades, the World Health Organization estimates that more than 125 million workers worldwide are regularly exposed to asbestos, leading to approximately 107,000 deaths annually [118]. Moreover, exposure to carbon nanotubes (CNTs), used in the manufacture of televisions, tennis rackets, sports cars, and computer motherboards has also been identified as a potential risk factor for mesothelioma [119,120].

**Table 1:** Summary of the cancer mouse models tested for si-VDAC1.

	Cell Line/Carcinogen	siRNA, Concentration & RNAi Delivery Means	Mouse Model	Administration	Refs.
1	Hela	sh-VDAC1, Metafectene	Cervical cancer, SC	Intertumoral	[121]
2	A549	si-hVDAC1, 50 nM, Jet-Prime	Lung cancer, SC	Intertumoral	[75,94]
3	U87-MG	si-hVDAC1, 50 nM	GBM, SC	Intertumoral	[74,94,122]
4	U87-MG	PLGA–PEI-si-hVDAC1, 200 nM	GBM Intracranial-orthotopic	Intravenous	[74]
5	U118-MG	si-hVDAC1, 50 nM, Jet-Prime	GBM, SC	Intertumoral	[74]
6	MZ-18, PDX	PLGA-PEI-si-hVDAC1, 200 nM	GBM Intracranial- orthotopic	Intravenous	[74]
7	MDA-MB-231	si-hVDAC1, 50 nM, Jet-Prime	Breast cancer, SC	Intertumoral	[94]
8	HepG2	si-hVDAC1, 50 nM, Jet-Prime	Hepatocellular carcinoma, SC	Intertumoral	[75]
9	H226	si-m/hVDAC1, 50 nM, Jet-Prime	Mesothelioma cancer, SC	Intertumoral	[123]
11	Urethane	PLGA-PEI- si-m/hVDAC1-B, 200 nM	Chemically induced-lung cancer	Intravenous	[105]
12	UM-UC-3	si-m/hVDAC1, 50 nM, and 200 nM, Jet-Prime	Bladder cancer, SC	Intertumoral	[116]
13	N-butyl-N-(4-hydroxy-butyl) nitrosamine	PLGA-PEI si-m/hVDAC1, 50 nM	Chemically induced-bladder cancer	Intravesical	[116]
14	H-69	si-m/hVDAC1, 50 nM, Jet-Prime	Small lung cancer, SC	Intertumoral	[105]

In our published study and using si-m/hVDAC1, we found that silencing VDAC1 expression led to significant changes in cancer cell metabolism. This treatment markedly inhibited mesothelioma cell and tumor growth, and effectively eliminated cancer stem cells (CSCs) [123]. Moreover, si-VDAC1 treatment prompted the differentiation of pluripotent malignant mesothelioma cells, thereby reducing the malignancy of the tumors [123].

## 6. VDAC1-Depletion Modulating the Tumor Microenvironment (TME)

The TME is a complex and dynamic network encompassing cancer cells, stromal tissues (such as immune cells, fibroblasts, myofibroblasts, cytokines, and vascular tissues), and the ECM [124].

Various cell types within the TME have been studied for their roles in tumor progression and as potential targets for chemotherapy. For instance, tumor-infiltrating lymphocytes (TILs), which include immune thymocyte (T) cells, B lymphocytes (B) cells, and natural killer cells (NKs), have been shown to significantly impact tumor prognosis. The density of CD8^+^ T cells, in particular, is associated with improved disease-free survival, disease-specific survival, and overall survival [125].

The TME also contains myeloid populations that infiltrate the tumor and contribute to its progression. This includes myeloid-derived suppressor cells (MDSCs), dendritic cells (DCs), and tumor-associated macrophages (TAMs). MDSCs are a heterogeneous group of cells characterized by their myeloid origin, immature state, and ability to suppress T-cell responses, which promotes tumor maintenance, progression, and resistance to anticancer therapies [126].

Among the prominent non-cancerous cell types in the TME are fibroblasts. These cells are non-vascular, non-epithelial, and non-inflammatory components of connective tissue. Located within the fibrillar matrix, fibroblasts are crucial for its synthesis and maintenance. They play essential roles in the deposition of the ECM, and regulation of epithelial differentiation and modulation of inflammation, and they are involved in wound healing [127]. Fibroblasts are responsible for producing various ECM components, including collagen types I, III, and V, as well as fibronectin. They also contribute to basement membrane formation through the secretion of type IV collagen and laminin. Additionally, fibroblasts are a key source of ECM-degrading proteases, such as matrix metalloproteinases (MMPs), underscoring their vital role in maintaining ECM homeostasis [128]. They infiltrate lesions, create ECM scaffolds for other cells, and contain cytoskeletal elements that aid in wound contraction and healing [128].

It is well-established that the TME plays a critical role in cancer progression, significantly influencing the spread of tumor cells to secondary sites through complex interactions between cancer cells and surrounding stromal cells [129]. Our research has shown that treatment with si-VDAC1 leads to notable changes in the TME across various tumor types (Figure 3) [74,104,116]. We further studied the impact of silencing VDAC1 on the TME using human-derived A549 lung cancer xenografts in mice. Using immunofluorescence staining of key TME markers and next-generation sequencing (NGS) to distinguish between human and mouse gene origins allowed for a clear analysis of the interactions between the TME and malignant cells [104].

Our findings, as indicated above, show that depleting VDAC1 in cancer cells led to metabolic reprogramming, tumor regression, and disruption of tumor–host interactions [74,104,116]. The effects are reflected in the altered expression of genes related to TME, including those involved in ECM organization, matrix-related peptidases, angiogenesis, intercellular interactions, integrins, and growth factors linked to stromal functions [104]. Furthermore, silencing VDAC1 in cancer cells impacted the expression of structural proteins, matrix metalloproteinases, and lysyl oxidase (Lox) [104], triggering a stromal response similar to that observed during wound healing. The results reflect the complex remodeling of the TME by cancer cells in the tumor and suggest that cancer metabolic reprogramming via VDAC1 depletion targets both the cancerous and stromal compartments within a tumor—a relation that may offer therapeutic potential.

## 7. VDAC1-Depletion and Stem Cells

CSCs are a subset of cancer cells with characteristics similar to normal stem or progenitor cells, including self-renewal and multi-lineage differentiation capabilities [130]. These cells drive tumor growth and contribute to tumor heterogeneity by regenerating themselves, differentiating into various cell types, and expressing genes typically found in embryonic and somatic stem cells. CSCs are notably resistant to conventional cancer therapies [131].

Unlike normal stem cells, CSCs are not regulated in the same manner, which allows them to proliferate uncontrollably and to sustain tumor growth [130]. CSCs, which constitute approximately 1–2% of the total cell population in various cancers, have been identified in tumors of the breast, lung, colon, brain, head and neck, prostate, liver, and other organs [131].

Our previous study demonstrated that reducing VDAC1 expression in GBM and breast cancer led to comprehensive changes in cancer hallmarks [74,94]. We found that si-VDAC1 treatment effectively inhibited tumor growth in both subcutaneous and intracranial-orthotopic GBM mouse models. Moreover, residual tumor cells exhibited a reversal of multiple oncogenic traits, including stemness, and differentiated into cell types resembling neurons and astrocytes [74,94,122].

The effects of VDAC1 depletion were linked to alterations in TFs that regulate signaling pathways associated with cancer hallmarks. Targeting VDAC1, which bridges metabolism and oncogenic signaling networks, resulted in the differentiation of CSCs into neuron-like cells [74,94,122].

Similarly, in MDA-MB-231 cells, which do not express ER, PR, or ERBB2/Her2, si-hVDAC1 treatment of MDA-MB-231 cells [93] or tumors derived from these cells [94] led to the expression of prolactin, ER and PR. and ERBB2/Her2, suggesting differentiation into less malignant lineages with si-hVDAC1 treatment.

A recent study [116] showed that suppressing the expression of VDAC1 in BC, using both subcutaneous and chemically-induced mouse models, resulted in a reduction of stemness characteristics associated with CSCs. This was evidenced by a decrease in expression levels of markers such as cytokeratin-14, ALDH1a, and the transcription factors SOX2 and CD44 [116].

In summary, depleting VDAC1 effectively targeted radio- and chemo-resistant CSCs in GBM, breast cancer, and BC, leading to their differentiation into mature, non-replicating cells and, thereby, preventing tumor recurrence.

## 8. microRNA-Mediated Regulation of VDAC1

MicroRNAs (miRNAs) play a crucial role in regulating a wide range of biological processes and are implicated in the development of numerous diseases. Research has shown that different categories of miRNAs significantly impact numerous health issues, including cancer [132]. Several miRNAs that target VDAC1 have been identified and are found to be altered under pathological conditions. For example, miR-34a and miR-29a, which are downregulated in hepatocellular carcinoma and ovarian cancer, play a critical role in apoptosis by targeting the 3′-UTR of VDAC1 mRNA [133]. Additionally, modulation of miR-7a-5p, known to regulate VDAC1 levels, has been reported to reduce hypoxia/reoxygenation-induced cardiomyocyte apoptosis [134]. Other miRNAs involved in regulating VDAC1 expression include miR-320a and lncRNA-H19/miR-675 [133,135], and miR-7, was reported to inhibit VDAC1 levels, cancer cell survival and metastasis in hepatocellular carcinoma [136], potentially by affecting the mitochondrial permeability transition pore [137].

Numerous miRNAs have been and are being evaluated for therapeutic applications across several disease categories, including cancer therapy. MRX34, a miR-34a mimic, was evaluated as an anticancer therapy in two phase II clinical studies for primary liver cancer, SCLC, lymphoma, melanoma, multiple myeloma, renal cell carcinoma, and NSCLC [138].

Given the association of high VDAC1 expression with various pathological conditions, it is evident that specific miRNAs capable of modulating VDAC1 levels have potential as therapeutic agents.

## 9. VDAC1-Depletion Affects Epigenetic-Related Enzymes and Substrates

Reprogramming cancer cells to counteract all hallmark features of tumors is most likely mediated via epigenetic considerations, with the interplay between metabolism and epigenetics being well-documented [139,140,141,142,143,144,145,146,147,148,149]. Epigenetic mechanisms play a vital role in cellular phenotypic plasticity. Disruptions in the regulation of epigenetic-related enzymes expression and, substrates, can lead abnormal cell fate determination, creating a pathway for oncogenic transformation. The metabolic state and chromatin structure are tightly linked, enabling cells to adapt gene expression and metabolism in response to environment alterations, often through epigenetically-driven processes [150]. Many enzymes involved in epigenetic gene regulation rely on co-substrates produced by cellular metabolism, highlighting a potential link between nutrition, metabolism, and gene regulation. This metabolism–epigenetics connection has been extensively explored in the context of tumorigenesis [142,147].

As a transporter of metabolites, VDAC1 plays a crucial role in regulating metabolic and energy homeostasis, significantly influencing the metabolic phenotype of cancer cells [151]. Our research showed that targeting VDAC1 with si-VDAC1 led to reprogramming of cancer metabolism, which in turn reduced tumor growth and invasiveness, inhibited angiogenesis, eliminated CSCs, and promoted tumor cell differentiation [74].

Silencing VDAC1 expression has been shown to cause significant changes in histone modifications, with increased histone acetylation and decreased histone methylation [122]. Histone acetylation is regulated by the opposing activities of histone acetyltransferases (HATs) and histone deacetylases (HDACs). In tumors treated with si-hVDAC1, there was a notable increase in the expression of acetyltransferases lysine acetyltransferase 2A, 5, and 7 (KAT2A, 5, 7), while the expression of acetyltransferase KAT2B, which promotes cell proliferation and transcriptional activation, and deacetylase HDAC2, which is linked to tumor de-differentiation and invasion, was highly reduced [122].

Histone acetylation reduces the electrostatic attraction between histone proteins and DNA, promoting a chromatin structure that facilitates gene transcription [152]. This is in line with the observed changes in the altered expression of over 4500 genes [74].

In tumors treated with si-hVDAC1, there was an increase in the expression of the protein deacetylases SIRT1 and SIRT6. SIRT1 is associated with metabolic homeostasis through its role in deacetylating target proteins [153], while SIRT6 regulates several critical pathways via epigenetic mechanisms, primarily through histone deacetylation. Thus, the high increase in SIRT-1 and SIRT-6 expression in si-hVDAC1-treated tumors underscores their role in the metabolism–epigenetics link, as sirtuins are NAD^+^-dependent deacetylases [122].

These findings illustrate the interaction between metabolic reprogramming and epigenetic changes induced by VDAC1 silencing, highlighting a novel area in cancer biology that could pave the way for new anti-cancer therapeutic strategies.

To summarize, the above findings propose that the depletion of VDAC1 in cancer cells disrupts the production and exchange of metabolites between the mitochondria and the rest of the cell, resulting in metabolic reprogramming of cancer cells [74,93,94]. Additionally, the effects of VDAC1 depletion involve changes in the levels of transcription factors that regulate signaling pathways associated with key cancer hallmarks [74,93,94]. The interplay between the metabolism and epigenetics is disrupted by VDAC1 depletion, resulting in changes to histone modifications, particularly in their methylation and acetylation states. Furthermore, these epigenetic modifications result in changes in the expression of over 4000 genes, including those related to epigenetic regulation [74,122]. These changes can account for the metabolic rewiring of the malignant cancer phenotype and the reversal of the tumor oncogenic properties.

## 10. Exploring the Role of VDAC1 Expression in Diseases Other than Cancer Using siRNA Targeting VDAC1

VDAC1 is overexpressed in a variety of diseases, reflecting its significant role in disease pathology. This overexpression is observed in cancer [1,19,54,75,121,154], Alzheimer’s disease (AD) [65,155,156,157], type 2 diabetes (T2DM) [64,158,159], autoimmune diseases such as lupus [62], cardiovascular diseases [160,161,162,163,164,165,166,167,168], inflammatory bowel diseases (IBDs) [61], non-alcoholic fatty liver disease (NAFLD) [53], COVID-19 [70], viral associated diseases [169,170,171,172,173,174,175,176,177], spinal cord injury [68], kidney disease [178].

VDAC1 overexpression leads to the activation of cell death pathways. In cancer cells, VDAC1 overexpression supports increased metabolite and energy supply. However, cancer cells also overexpress anti-apoptotic proteins to counteract VDAC1-mediated cell death [179]. In multiple diseases, VDAC1 overexpression correlates with disease progression, and reducing this expression has been shown to modulate disease symptoms, highlighting its potential as a therapeutic target.

### 10.1. VDAC1 Expression Levels and Cardiovascular Diseases

The role of VDAC1 in the pathogenesis of cardiac abnormalities has been well documented. In the context of cardiac ischemia/reperfusion, increased VDAC1 expression and phosphorylation have been linked to enhanced cardiomyocyte damage, with inhibition of these processes associated with the nutritional pre-conditioning effects of resveratrol [163,165,166,167]. Oxidative stress-induced damage in H9c2 myoblasts has been shown to elevate VDAC1 expression and promote its oligomerization [161,168]. Upregulated transcriptional levels of a diverse array of genes including VDAC1 were found in the septal tissue of human patients with hypertrophic cardiomyopathy [164]. Recently, we found increased VDAC1 levels in various cardiac pathologies, including post-myocardial infarction, chronic left ventricular dilatation and dysfunction, and hyperaldosteronism [162]. Additionally, VDAC1 expression was significantly upregulated in a rat model of cardiac hypertrophy induced by renal artery ligation, and treatment with siRNA against VDAC1 partially mitigated apoptotic cell death [180]. VDAC1 expression was downregulated in a cellular model of cardiomyocyte hypertrophy induced by the α1-adrenergic agonist phenylephrine, an effect that was prevented by peroxisome proliferator-activated receptor-α (PPARα) [181].

Furthermore, silencing VDAC1 expression using lncRNA-H19/miR-675 has been reported to regulate high glucose-induced apoptosis by targeting VDAC1, suggesting a novel therapeutic strategy for diabetic cardiomyopathy [182].

The expansion of siRNA-based cardiovascular therapeutics is anticipated to make a significant impact in the near future.

### 10.2. VDAC1 Expression Levels and Neurodegenerative Diseases

Mitochondrial dysfunction and oxidative stress have been observed in postmortem AD brains [183,184,185,186,187,188], amyloid-β-precursor protein transgenic mice [189,190,191,192,193,194,195], cells expressing mutant APP [190,196,197,198], and cells treated with amyloid-β (Aβ) [199,200,201,202,203]. VDAC1 is overexpressed in post-mortem brains of AD patients and transgenic mice [65,155,156]. VDAC1 plays a role in Aβ-induced toxicity [204], with its presence on the plasma membrane promoting cell death, and it enhances Aβ-mediated apoptosis [205]. Silencing VDAC1 expression inhibited the entry of Aβ into the cytosol and reduced Aβ-induced toxicity [28]. RNA-mediated silencing VDAC1 expression in SHSY-5Y cells resulted improvement in synaptic activity and mitochondrial function [206].

Recently [65], we demonstrated that Aβ-induced VDAC1 overexpression in primary cultured neurons, and in an AD-like 5XFAD mouse model that VDAC1 was found to be overexpressed in neurons surrounding Aβ plaques, and this is associated with neuronal cell death. VBIT-4 has been shown to prevent AD-related pathophysiological changes, including neuronal cell death, neuroinflammation, and neuro-metabolic dysfunctions, and to mitigate cognitive decline in 5XFAD mice. The study suggests that targeting mitochondrial dysfunction through VDAC1, with VBIT-4 as a potential therapeutic agent, offers a promising strategy for modifying AD [65].

Scopolamine, a nonselective antagonist of muscarinic cholinergic receptor, induces memory loss by disrupting synaptic plasticity and altering associated gene expression, leading to amnesia. In the hippocampus of scopolamine-induced amnesic mice, VDAC1 levels were significantly reduced. Silencing VDAC1 expression in the hippocampus of healthy young mice resulted in impaired recognition memory. These findings suggest that downregulation of VDAC1 may contribute to neurodegeneration through mitochondrial disintegration in the hippocampus of scopolamine-induced amnesic mice [207,208].

To treat neurodegenerative diseases with siRNAs, overcoming the blood–brain barrier is crucial. This challenge can be addressed using nanocarriers. It is anticipated that the development of siRNA-based gene knockdown therapies will lead to several candidates, advancing to clinical trials in the near future [209].

### 10.3. VDAC1 Involvement in Viral and Bacterial Infections Was Demonstrated Using Specific siRNA Targeting VDAC1

During pathogen-host coevolution, viruses have developed various strategies to evade the host’s biochemical and immunological defenses, including the regulation of apoptosis, inflammation, and immune responses. Viruses manipulate mitochondrial function to affect energy production, metabolism, and innate immune signaling [210,211]. Viruses can modulate apoptosis in diverse ways and across different tissue types [212,213].

Several studies have shown that some viruses induce VDAC overexpression or interact with VDAC through their proteins [169,170,171,172,173,174,175,176,177]. Silencing VDAC expression has been found to significantly reduce viral protein levels. For example, the viral capsid protein Orf3 of hepatitis E virus (HEV) enhances VDAC1 levels and induces apoptosis in infected cells [171,172].

Reducing VDAC1 expression with siRNA diminished cell survival, highlighting VDAC1’s role in HEV pathogenesis [172]. In the case of dengue virus (DENV) infection, VDAC involvement was assessed by downregulating VDAC levels with siRNA. The DENV E protein interacts with the chaperone GRP78, which in turn associates with VDAC1 [174]. Downregulation of VDAC by siRNA reduced the expression of DENV non-structural proteins (NS1, NS3, NS5) [174]. The infectious bursal disease virus (IBDV) causes immunosuppressive disease in young chickens, upregulates VDAC1 expression in cells, and the IBDV protein VP5 induces apoptosis via interaction with VDAC. Additionally, silencing VDAC1 decreased the levels of viral proteins (VP1, VP2, and VP5) [177]. These results underscore the significant role of VDAC in viral infection.

*Mycobacterium avium* (*M. avium*) infection in humans primarily acts as a pulmonary pathogen in humans, affecting immunocompromised individuals such as those with AIDS and hairy cell leukemia, or patients undergoing immunosuppressive chemotherapy and with chronic lung disease. VDAC has been identified as a component of *M. avium* vacuoles within macrophages. Silencing VDAC expression with specific siRNA significantly reduced *M. avium* survival. This decrease is attributed to VDAC’s function in exporting bacterial cell wall lipids from the vacuole, suggesting that targeting VDAC could be a promising therapeutic strategy for infectious diseases [214].

### 10.4. VDAC1 Involvement in Other Diseases, as Demonstrated Using Specific siRNA Targeting VDAC1

5-aminolevulinic acid-mediated sonodynamic therapy (ALA-SDT) induces intracellular reactive oxygen species generation, loss of mitochondrial membrane potential, and apoptosis in THP-1 macrophages. Downregulation of VDAC1 expression using siRNA significantly reduced cell apoptosis, highlighting VDAC1′s crucial role in ALA-SDT-induced apoptosis in THP-1 macrophages. These findings suggest that targeting VDAC1 could be a viable strategy for regulating macrophage apoptosis, with potential therapeutic implications for atherosclerosis [215].

The therapeutic potential of siRNA targeting VDAC1 is evident given the association of VDAC1 overexpression with various pathological conditions, as presented above.

## 11. siRNA-VDAC1 Delivery: Experimental Evidence, Challenges, and Future Directions

Given the association between VDAC1 overexpression and various diseases, as discussed in Section 10, silencing VDAC1 expression with specific siRNA is expected to significantly impact the treatment of the VDAC1 that is overexpressed pathological conditions. Numerous studies we and others have shown that silencing VDAC1 using siRNA and transfection agents such as Lipofectamine 2000, [206], and JetPRIME or metafectene (Table 1), or X-tremeGENE [215], RNAiMAX [177] or Continuum transfection reagent [214] leads to reduced cell viability in various cancer cell lines.

In pre-clinical studies utilizing animal models, delivering siRNA that targets VDAC1 is crucial for protecting it from degradation and for enhancing its therapeutic efficacy. For instance, some studies involved directly injecting naked siRNA against VDAC1 into the ventricles of aortic-ligated rats [180]. However, most studies employed delivery systems for the siRNA. In cancer research, nanoparticles composed of biodegradable polymers, such as poly(lactic-co-glycolic acid) (PLGA) and polyethyleneimine (PEI), were used for encapsulation (Table 1). We have shown using this delivery system, in which, siRNA-VDAC1 successfully targeted the brain, lungs, and bladder, resulting in a significant reduction in tumor growth, metabolic reprogramming, and a reversal of tumor properties [74,105,116].

It is important to explore additional delivery systems for siRNA targeting of VDAC1. These include exosomes, which are naturally occurring biocompatible extracellular vesicles that can carry siRNA and facilitate targeted delivery to recipient cells, enhancing cellular uptake through their native interactions with cell membranes [216]. Other options are positively charged liposomes that bind to negatively charged siRNA, forming complexes capable of penetrating cell membranes, and lipid-based nanoparticles (LNPs) that facilitate siRNA uptake via endocytosis [217] as well as others that are presented in the next section [218,219,220].

Given VDAC1’s role in various diseases, targeting it presents a promising strategy for treatment. Although direct clinical trials specifically targeting VDAC1 remain limited, ongoing research in RNA interference (RNAi) is paving the way for translating siRNA-VDAC1 from laboratory studies to clinical applications.

## 12. siRNA as a Therapeutic Agent: Challenges, Potential and Clinical Development

RNA interference (RNAi) involves introducing synthetic siRNA into target cells, where the siRNA’s complementary nucleotide sequences lead to degradation of mRNA. This process prevents translation and effectively silences the gene(s) of interest. Gene silencing using specific siRNA can target any gene, including those considered ‘undruggable’ by small molecules or protein-based drugs, offering a potential solution for challenging therapeutic targets [221,222,223,224].

Synthetic siRNAs have been studied as treatments for a range of human diseases, including cancers, viral infections, eye conditions, genetic disorders, and cardiovascular diseases. Although siRNAs have shown promising therapeutic efficacy in vivo, several significant challenges persist, particularly related to stability, delivery and efficiency that should be overcome. Specifically, these issues—often referred to as the three ‘E’ challenges—that are crucial for advancing siRNA therapies into clinical practice [224]. These are Entry—targeting siRNA for effective cellular uptake and accumulation, Escape from being trapped in endosomes/lysosomes, where it may be degraded by enzymes and Efficacy—achieving in vivo efficacy characterized by good stability, long-lasting effects, and safety in pharmaceutical performance [224].

Some of these limitations have been addressed through the following strategies:

Delivery: A primary challenge in pre-clinical studies is effectively delivering siRNA to target cells and tissues. Due to their relatively large size and negative charge, siRNA molecules struggle to cross cellular membranes. To overcome this, various non-viral delivery systems have been developed to facilitate cellular uptake and protect the RNA from degradation. These systems include lipid nanoparticles (LNPs), polymer-based carriers, inorganic nanoparticles, and exosome-based delivery systems. Despite advancements, issues related to loading capacity, stability, safety, and efficacy of these delivery systems persist [218,219,220].

Each of these delivery systems has its own advantages and challenges. The choice of a specific system often depends on factors such as the type of cells being targeted, the therapeutic context, and the desired efficiency and safety profile of the siRNA therapy. Ongoing research continues to refine these delivery methods to optimize the therapeutic potential of siRNA targeting of VDAC1.

Stability: One of the major challenges in using siRNAs as therapeutic agents is their poor stability. siRNA molecules are highly susceptible to degradation by serum and cellular nucleases. To address this, encapsulation and chemical modifications strategies have been developed to protect siRNAs from nuclease degradation and, thereby, enhance their stability in vivo [221].

Immune Responses: siRNAs can trigger immune responses both in a sequence-independent and sequence-dependent manner, potentially leading to inflammation or other adverse effects. To ensure the safe use of siRNA therapeutics, it is crucial to understand and mitigate these immune reactions. Chemical modifications of the siRNA duplexes have been shown to reduce immunogenicity [225].

Off-Target Effects: siRNA can sometimes bind to and silence unintended mRNA targets, leading to off-target effects. To minimize these effects, it is crucial to design siRNAs with high specificity and to validate their target interactions. Strategies to reduce off-target effects include using the lowest possible siRNA concentrations, as these effects are concentration-dependent [226]. Additionally, chemical modifications of the siRNA have been shown to reduce off-target effects.

Thus, chemical modification of siRNAs can improve their stability, minimize immunogenicity, and reduce off-target effects of siRNAs, enhancing their overall therapeutic efficacy.

While small molecules have been developed for over a century and proteins and antibodies have been studied for nearly 50 years, nucleic acid molecules have emerged as a novel therapeutic approach only in the past 20–30 years. Despite their relatively recent development, they have already garnered significant global interest from the pharmaceutical industry and are regarded as highly promising therapeutics.

In recent years, siRNA therapy has shown immense potential in the development of numerous candidate drugs for preclinical and clinical research, with cancer currently the major target of siRNA therapeutics [222,227]. There are six siRNA therapeutics that have been approved for clinical use, and approximately 20 additional candidates have progressed to the late stages of clinical investigation, and globally 15 investigational siRNA drugs are in clinical Phase 2 or later stages (as of August 2023) [224].

Currently, there are very few small molecules that specifically target VDAC1, such as VBIT-4 and VBIT-12 [56], which prevent its oligomerization. Other molecules, like clotrimazole, bifanazole [228], and methyl jasmonate [229] target its interactions with its associated protein HK. Notably, VDA-1102, which targets the VDAC1-HK2 interaction, is the only small molecule currently in clinical trials for actinic keratosis (VIDAC Pharma). The advantages and disadvantages of siRNA compared to small molecules are not unique to VDAC1 but are generally applicable. siRNA silencing provides high specificity for target mRNA degradation, which results in precise gene knockdown, while minimizing off-target effects on other genes. In contrast, small molecules may lack selectivity and may lead to off-target effects. The disadvantages of siRNA include challenges with delivery and the potential to trigger immune responses, these risks can be significantly reduced with modifications. On the other hand, small molecules are easier to deliver and can offer sustained effects although they carry a higher risk of off-target impacts. In summary, siRNA silencing excels in specificity and targeted gene knockdown, while small molecules offer convenience and longer-lasting effects, albeit with increased risks of off-target interactions.

The siRNA therapeutics that have been approved for clinical use (none for cancer) include: patisiran (Onpattro) targeting transthyretin (TTR) mRNA was approved in 2018 for the treatment of hereditary transthyretin-mediated amyloidosis (hATTR amyloidosis); givosiran (Givlaari targeting aminolevulinic acid synthase 1 (ALAS1) mRNA to decrease the production of neurotoxic heme precursors was approved in 2019 for the treatment of acute hepatic porphyria (AHP); lumasiran (Oxlumo), targeting hydroxyacid oxidase 1 (HAO1) mRNA to lower oxalate production was approved in 2020 for the treatment of primary hyperoxaluria type 1 (PH1); and inclisirani (Leqvio), targeting PCSK9 mRN to reduce low-density lipoprotein (LDL) cholesterol levels was approved in 2021 for the treatment of hyperlipidemia. In addition, two siRNA therapeutics that are currently in the advanced stages of development are: DCR-PH1, targeting primary hyperoxaluria, and DCR-HBVS, focusing on silencing viral proteins in hepatitis B.Alongside the approved siRNA treatments mentioned above, several new ones are in clinical trials, and five treatments have advanced to the critical Phase III trials. These are: fitusiran, nedosiran, teprasiran, tivanisiran, and vutrisiran [138].

## 13. Conclusions

In this review, we illustrate how metabolic reprogramming of cancer cells triggers a series of events that lead to changes in multiple cancer hallmarks (Figure 3). Depletion of VDAC1 results in metabolic reprogramming, which subsequently regulates epigenetically-related enzymes and gene transcription. This effect is observed in different biological contexts and contributes to tumor reprogramming, including growth inhibition. The reprogramming process induced by VDAC1 depletion is dynamic over time, affecting both cells in culture [93] and tumors [74,94]. This is reflected in the downregulation of proteins such as metabolic enzymes, CSCs markers, and certain transcription factors, as well as the upregulation of proteins like p53 and those associated with cell differentiation and epigenetics. This reciprocal relationship highlights a connection between decreased cellular metabolism, reduced cell growth and stemness, and induced differentiation, as seen in both cultured cells and tumor models, including their microenvironments.

The results for cancer and other diseases underscore RNAi as a powerful tool for modulating VDAC1 expression, which is frequently overexpressed in these conditions. Challenges such as off-target effects, the instability of naked siRNA, and susceptibility to nucleases have been addressed by developing advanced delivery systems. Given the research advancements and successes over the past two decades, siRNA-based therapies are expected to see greater clinical application. With their specificity, cost-effectiveness, and the use of nanocarriers, it is anticipated that more siRNA-based knockdown targets will move into clinical trials in the near future.

## Data Availability

Not applicable.
